# Efficacy and Safety of Aprocitentan in the Treatment of Hypertension: A Meta-Analysis of Evidence from Randomized Controlled Trials

**DOI:** 10.31083/RCM25909

**Published:** 2025-01-20

**Authors:** Li Zheng, Ming Liu, Xiaotong Gu, Yatong Zhang, Yan Wang

**Affiliations:** ^1^Department of Pharmacy, China Aerospace Science & Industry Corporation 731 Hospital, 100074 Beijing, China; ^2^Evidence-Based Medicine Center, School of Basic Medical Sciences, Lanzhou University, 730000 Lanzhou, Gansu, China; ^3^Department of Pharmacy, Beijing Hospital, National Center of Gerontology, Institute of Geriatric Medicine, Chinese Academy of Medical Sciences, 100730 Beijing, China; ^4^Department of Cardiovascular Medicine, Beijing Hospital, National Center of Gerontology, Institute of Geriatric Medicine, Chinese Academy of Medical Sciences, 100730 Beijing, China

**Keywords:** hypertension, dual endothelin receptor antagonist, aprocitentan, meta-analysis

## Abstract

**Background:**

Hypertension is one of the most prevalent disorders encountered in medical practice, yet effective pharmacotherapy options for resistant hypertension are limited. In this meta-analysis, we aimed to evaluate the efficacy and safety of aprocitentan in treating hypertension.

**Methods:**

We searched PubMed, Embase, ClinicalTrials.gov, and the Cochrane Library databases from inception to June 3, 2024, for randomized controlled trials (RCTs) that compared the efficacy and safety between aprocitentan and placebo in treating hypertension. According to the dosage of aprocitentan, the study was divided into a low-dose group (10–12.5 mg), medium-dose group (25 mg), and high-dose group (50 mg).

**Results:**

This meta-analysis included five RCTs, which incorporated 1224 patients, and displayed that aprocitentan can reduce the mean sitting systolic blood pressure (msSBP) [(low dose subgroup: mean difference (MD): –3.85 mmHg; 95% confidence interval (CI): –7.47 to –0.23; *p* = 0.040; medium dose group: MD: –5.56 mmHg; 95% CI: –10.69 to –0.44; *p *= 0.030)], mean sitting diastolic blood pressure (msDBP) (low dose subgroup: MD: –3.95 mmHg; 95% CI: –4.06 to –3.85; *p *< 0.001; medium dose group: MD: –4.75 mmHg; 95% CI: –5.91 to –3.60; *p *< 0.001), 24-hour ambulatory systolic blood pressure (maSBP) (low dose group: MD: –4.18 mmHg; 95% CI: –4.32 to –4.04; *p *< 0.001; medium dose group: MD: –5.89 mmHg; 95% CI: –6.03 to –5.75; *p *< 0.001), and 24-hour ambulatory diastolic blood pressure (maDBP) (low dose group: MD: –4.33 mmHg; 95% CI: –4.42 to –4.24; *p *< 0.001; medium dose group: MD: –5.82 mmHg; 95% CI: –5.91 to –5.73; *p *< 0.001). In the high-dose group, there was no difference between the aprocitentan and placebo groups in the msSBP (MD: –4.83 mmHg; 95% CI: –11.44 to 1.79; *p *= 0.150). Meanwhile, the safety profile of aprocitentan was good, and no significant differences in the frequency of adverse events (AEs) and serious adverse events (SAEs) were observed compared to the placebo.

**Conclusions:**

Aprocitentan significantly reduces blood pressure and has a good safety profile. However, it is worth noting that high doses of aprocitentan (50 mg) did not yield better blood pressure-lowering effects.

## 1. Introduction

Hypertension is a major contributing factor to cardiovascular diseases and deaths globally and has a prevalence in the total population of 30% to 40% [1, 2]. The health losses and economic burdens caused by hypertension and its associated complications may exceed the growth rate of the global population and economy, making hypertension an ongoing significant global public health issue [3]. Current guidelines recommend various classes of antihypertensive medications, including calcium channel blockers (CCBs), angiotensin-converting enzyme inhibitors (ACEIs), angiotensin II receptor blockers (ARBs), and beta-blockers. Based on National Health and Nutrition Examination Survey (NHANES) data, “Just under 70% of drug-treated hypertensive patients achieve blood pressure (BP) control below 140/90 mmHg, meaning over 30% still have elevated BP, and just under 60% fail to meet the target of <130/80 mmHg”. Challenges in hypertension treatment arise from patients exhibiting an intolerance to these drugs and the specific medication requirements for individuals with renal impairment. Hence, there is a need to develop antihypertensive medications with novel mechanisms of action [4].

One possible new target is the endothelin (ET) system, which exhibits increased activity under pathological conditions [5]. Endothelin-1 (ET-1) is a small peptide predominantly synthesized by vascular endothelial cells [6]. Additionally, ET-1 is a potent vasoconstrictor, a pathogenic factor in endothelial dysfunction, a growth factor, and a stimulant of aldosterone synthesis and catecholamine release [7]. ET-1 exerts its effects by acting on endothelin A (ETA) receptors in vascular smooth muscle cells and endothelin B (ETB) receptors in endothelial cells [8, 9, 10]. Overall, under physiological conditions, activation of ETA receptors leads to vasoconstriction, while activation of ETB receptors mediates vasodilation via nitric oxide release [10]. Studies have shown efficacy in blocking the ET-1 receptors in many models of hypertension, particularly under conditions of low-renin/salt-sensitive conditions [11, 12].

Aprocitentan is a potent and orally effective dual ET receptor antagonist that blocks the binding of ET-1 to ETA/ETB receptors [12]. Based on studies in rodent animal models, dual blockade of ETA/ETB receptors appears to carry a lower risk of fluid retention and vascular leakage compared to selective ETA blockade by excessive stimulation of ETB receptors, leading to non-selective vasodilation and vasopressin release [13]. Presently, the results of several randomized controlled trials (RCTs) have shown significant antihypertensive effects observed in clinical trials of aprocitentan, especially in patients with resistant hypertension.

This study aimed to conduct a meta-analysis, integrating the results of all published randomized controlled trials, to provide a more accurate assessment of the efficacy and safety of aprocitentan in the treatment of hypertension.

## 2. Methods

This systematic review and meta-analysis was performed according to the Cochrane Handbook and the Preferred Reporting Items for Systematic Review and Meta-Analysis (PRISMA) [14, 15].

### 2.1 Search Strategy

PubMed, Embase, the Cochrane Library, and ClinicalTrials.gov databases were searched from their inception to June 3, 2024, for articles using the following Medical Subject Headings or keywords: “aprocitentan”, “ACT–132577”, “Tryvio”, and “hypertension”. The detailed search strategy of all databases is presented in **Supplementary Text 1**. All published papers related to aprocitentan were searched. After completing the data extraction, we also updated the search to find the most recently published studies.

### 2.2 Eligibility Criteria

Trials that met the following criteria were included in this study: (1) the study was a RCT; (2) intervention was performed using aprocitentan; (3) changes in one or more of the following outcomes were measured: the changes were noted in the systolic blood pressure and diastolic blood pressure, while in the sitting position (mean sitting systolic blood pressure (msSBP), mean sitting diastolic blood pressure (msDBP)), changes were observed in the 24-hour ambulatory SBP (maSBP) and 24-hour ambulatory DBP (maDBP); (4) adverse events (AEs) or serious AEs (SAEs) were reported.

We excluded the studies presented as letters, case reports, reviews, conference abstracts, and articles with insufficient data. Additionally, animal experiments and studies that did not include relevant outcome indicators were excluded from the analysis.

### 2.3 Study Selection

All screening studies were performed using the Covidence software (Veritas Health Innovation, Melbourne, VIC, Australia; https://www.covidence.org/). Two authors (ML and XTG) independently screened the literature according to the titles and abstracts and excluded the studies that did not meet the inclusion criteria. The full text of the remaining articles was read to determine whether the study was eligible for inclusion in the analysis. When two authors disagreed, the decision was resolved by mutual consensus or adjudicated by a third author (YW).

### 2.4 Data Extraction

The information we collected included the study’s title, design, patient characteristics, interventions, outcome indicators (including msSBP, msDBP, maSBP, maDBP, AEs, and SAEs), and duration of treatment.

Two authors (LZ and XTG) independently extracted data. Disagreements were resolved through discussion or decided by a third author (YTZ). In the case of multiple records pertaining to the same study (e.g., original fulltext publication, abstract, and post-analysis), we collected and analyzed all relevant data as a single study.

### 2.5 Risk of Bias

All included studies were assessed using the Cochrane Risk of Bias Tool 2 (ROB2) [16] and based on six domains: bias arising from the randomization process, bias due to deviations from the intended intervention, bias due to missing outcome data, bias in measurement of the outcome, bias in selection of the reported result, and other biases. Two authors (LZ and ML) performed the risk of bias; all disagreements were resolved through discussion or adjudicated by a third author (YW).

### 2.6 Data Analysis

We performed this meta-analysis using the Review Manager (RevMan) 5.4.1 (Nordic Cochrane Centre, Copenhagen, Denmark). Dichotomous outcomes were assessed by risk ratios (RRs), while the mean difference (MD) was employed to express the continuous outcomes data, with 95% confidence intervals (CIs). The random-effects model was employed in this meta-analysis due to potential heterogeneity among the included studies. I^2^ was used to assess heterogeneity between included studies. An I^2^ value of <25% indicated low heterogeneity; 25% to 50% was moderate; >50% was high heterogeneity.

As none of the outcomes included more than 10 studies and small sample sizes per study, we did not explore sources of heterogeneity using meta-regression analysis. Sensitivity analysis was not planned. However, we used the magnitude of heterogeneity as one of the bases for the certainty of the evidence. We assessed the publication bias using the Egger test and funnel plots to determine if an outcome contained more than 10 studies.

## 3. Results

### 3.1 Study Selection

**Fig. 1.**
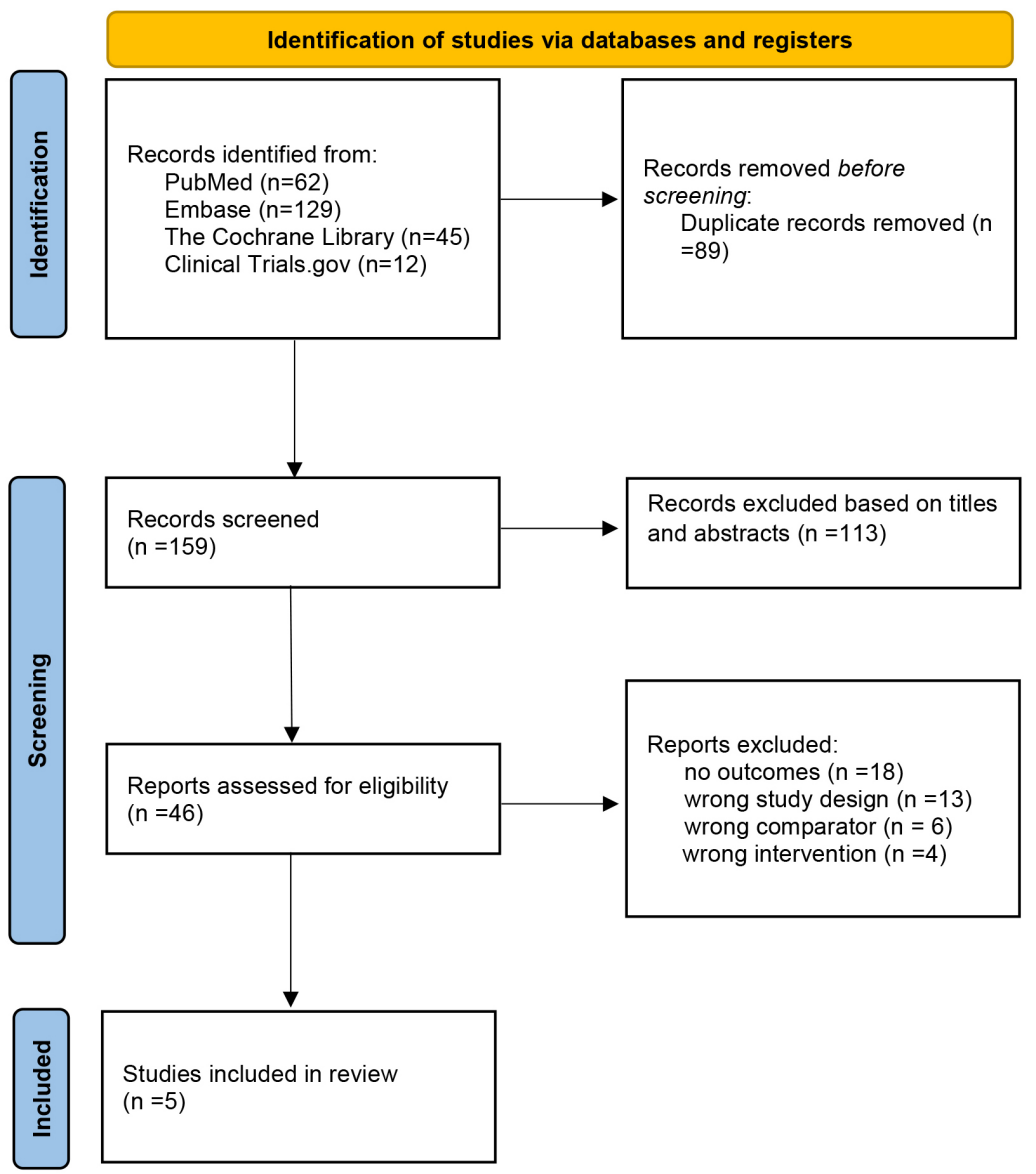
**The Preferred Reporting Items for Systematic Review and Meta-Analysis (PRISMA) flowchart of the study screen**.

A total of 248 studies were initially screened. Among them, 89 duplicate studies were automatically removed using EndNote software, and 113 were excluded based on their titles and abstracts. After reading the full texts, 41 studies were eliminated, leaving five studies [17, 18, 19, 20, 21] for inclusion in the final meta-analysis. The process of literature selection is shown in Fig. 1.

### 3.2 Study Characteristics

The five included studies [17, 18, 19, 20, 21] comprised 1224 patients with hypertension. Of these, 865 patients received aprocitentan, and 359 received a placebo. The age range of the included patients was 21–73 years old, and the range dose of treatment was 10 mg–50 mg (divided into low dose group: 10–12.5 mg; medium-dose group: 25 mg; high dose group: 50 mg). The detailed characteristics of the included studies are shown in Table 1 (Ref. [17, 18, 19, 20, 21]).

**Table 1.** **The detailed characteristics of the included studies in the meta-analysis**.

Study	Research design	Interventions	No.	Male	Mean age (y)	Duration of treatment
Schlaich *et al*. 2022 [17]	Multi-center, blinded, randomized, parallel-group, phase 3 trial	Aprocitentan 12.5 mg	243	144	61.2 ± 10.3	4 weeks
		Aprocitentan 25 mg	243	145	61.7 ± 10.4	
		Placebo	244	145	62.2 ± 11.2	
Gueneau de Mussy *et al*. 2021 [18]	Single-center, double-blind, randomized, placebo-controlled, two-way crossover study	Aprocitentan 10 mg	8	8	28.9	9 days
		Placebo	8	8		
		Aprocitentan 25 mg	7	7		
		Placebo	7	7		
		Aprocitentan 50 mg	8	8		
		Placebo	8	8		
Fontes *et al*. 2021 [19]	Single-center, double-blind, placebo controlled, randomized phase 1 study	Aprocitentan 25 mg	16	NR	NR	10 days
		Placebo	4			
Verweij *et al*. 2020 [20]	Randomized, double-blind, multicenter, placebo, and active comparator-controlled trial	Aprocitentan 10 mg	82	51	55.3 ± 9.8	8 weeks
		Aprocitentan 25 mg	82	45	55.1 ± 10	
		Aprocitentan 50 mg	81	53	54.2 ± 9.3	
		Placebo	82	55	53.5 ± 9.1	
Sidharta *et al*. 2019 [21]	Double-blind, randomized, placebo-controlled, parallel-group	Aprocitentan 25 mg	6	NR	NR	10 days
		Placebo	6			

Note: NR means not report.

### 3.3 Risk of Bias

Table 2 (Ref. [17, 18, 19, 20, 21]) displays the bias assessment for the studies included in this meta-analysis. One study [17] was determined to present a low probability of bias. Collectively, the studies included in this meta-analysis exhibited high quality, suggesting a minimal risk of bias.

**Table 2.** **The risk of bias of included studies**.

Study	Bias arising from the randomization process	Bias due to deviations from the intended intervention	Bias due to missing outcome data	Bias in the outcome measurement	Bias in the selection of the reported results	Other risk of bias	Overall judgement
Schlaich* et al*. 2022 [17]	Low	Probably low	Low	Low	Probably low	Low	Probably low
Gueneau de Mussy* et al*. 2021 [18]	Low	Low	Low	Low	Probably low	Low	Low
Fontes* et al*. 2021 [19]	Low	Low	Low	Low	Probably low	Low	Low
Verweij* et al*. 2020 [20]	Low	Low	Low	Low	Probably low	Low	Low
Sidharta* et al*. 2019 [21]	Low	Low	Low	Low	Probably low	Low	Low

### 3.4 Meta-Analysis

#### 3.4.1 Efficacy of Aprocitentan

**Fig. 2.**
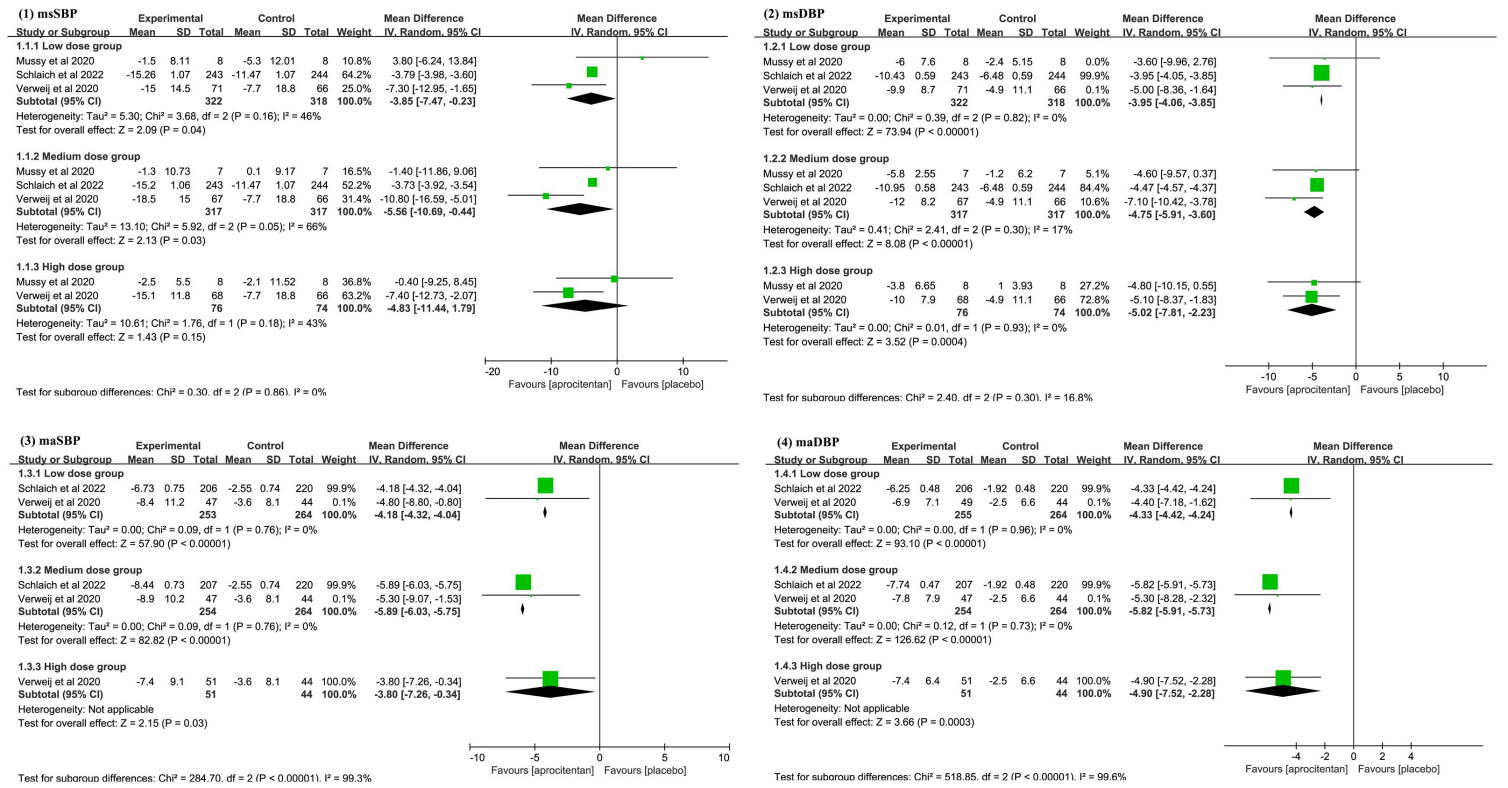
**Meta-analysis results on: (1) mean sitting systolic blood pressure (msSBP); (2) mean sitting diastolic blood pressure (msDBP); (3) mean 24-hour ambulatory systolic blood pressure (maSBP); (4) mean 24-hour ambulatory diastolic blood pressure (maDBP)**. CI, confidence interval; IV, inverse variance.

The subgroup analysis was performed according to the different doses of aprocitentan. Administering a low dose of aprocitentan treatment significantly reduced the msSBP (MD: –3.85 mmHg; 95% CI: –7.47 to –0.23; I^2^ = 46%; *p *= 0.040), msDBP (MD: –3.95 mmHg; 95% CI: –4.06 to –3.85; I^2^ = 0%; *p* < 0.001), maSBP (MD: –4.18 mmHg; 95% CI: –4.32 to –4.04; I^2^ = 0%; *p* < 0.001), and maDBP (MD: –4.33 mmHg; 95% CI: –4.42 to –4.24; I^2^ = 0%; *p* < 0.001). This indicates that a low dose of aprocitentan can significantly reduce blood pressure and affect 24-hour dynamic blood pressure. Treatment with a medium dose of aprocitentan also significantly reduced the msSBP (MD: –5.56 mmHg; 95% CI: –10.69 to –0.44; I^2^ = 66%; *p *= 0.030), msDBP (MD: –4.75 mmHg; 95% CI: –5.91 to –3.60; I^2^ = 17%; *p* < 0.001), maSBP (MD: –5.89 mmHg; 95% CI: –6.03 to –5.75; I^2^ = 0%; *p* < 0.001), and maDBP (MD: –5.82 mmHg; 95% CI: –5.91 to –5.73; I^2^ = 0%; *p* < 0.001). These data also indicate that the medium aprocitentan dose can significantly reduce blood pressure and affect 24-hour dynamic blood pressure. Treatment with a high dose of aprocitentan also significantly decreased the msDBP (MD: –5.02 mmHg; 95% CI: –7.81 to –2.23; I^2^ = 0%; *p *= 0.004), maSBP (MD: –3.80 mmHg; 95% CI: –7.26 to –0.34; I^2^: not applicable; *p *= 0.030), and maDBP (MD: –4.90 mmHg; 95% CI: –7.52 to –2.28; I^2^: not applicable; *p *= 0.003). However, both groups presented no significant difference in msSBP (MD: –4.83 mmHg; 95% CI: –11.44 to 1.79; I^2^ = 43%; *p *= 0.150). This indicates that the high dose of aprocitentan can significantly reduce 24-hour dynamic blood pressure and lower msDBP, but no significant effect was observed on the msSBP. The details can be seen in Fig. 2.

#### 3.4.2 Safety of Aprocitentan

**Fig. 3.**
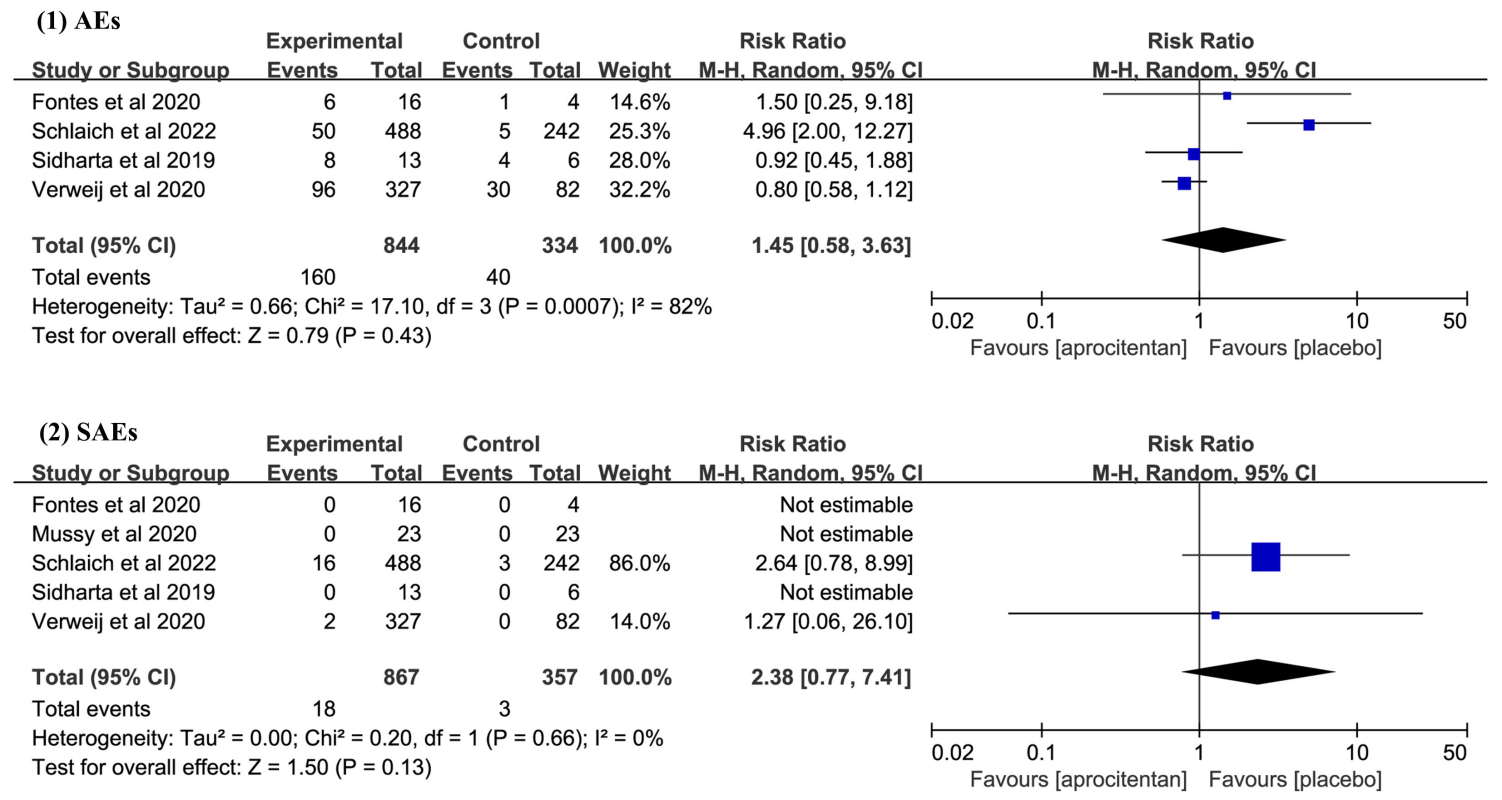
**Meta-analysis results on the incidence of adverse events (AEs) and serious adverse events (SAEs)**. CI, confidence interval; M-H, Mantel-Haenszel.

All included studies [17, 18, 19, 20, 21] reported data on patients with SAEs, with three studies [18, 19, 21] indicating no occurrence of SAEs. Four studies [17, 19, 20, 21] reported data on patients with AEs. The pooled results showed that there was no statistically significant difference between aprocitentan and placebo groups in the incidence of AEs (RR: 1.45; 95% CI: 0.58 to 3.63; I^2^ = 80%; *p *= 0.510, Fig. 3) and SAEs (RR: 2.38; 95% CI: 0.77 to 7.41; I^2^ = 0%; *p *= 0.130, Fig. 3).

## 4. Discussion

This meta-analysis investigated the efficacy and safety of aprocitentan for treating patients with hypertension. The main findings of this study were as follows: (1) low (10–12.5 mg) and medium doses (25 mg) of aprocitentan can significantly reduce msSBP, msDBP, maSBP, and maDBP, but a high aprocitentan dosage (50 mg) did not significantly reduce msSBP relative to placebo treatment. Notably, while the highest dose significantly reduced blood pressure statistically, there was no clear incremental benefit over the medium or low doses. This observation suggests a flattened dose-response curve, where higher doses do not proportionally increase efficacy; (2) there were no significant differences in the incidence of AEs and SAEs between both groups. In addition, it is worth noting that a high dose of aprocitentan (>25 mg) is absorbed slowly, and in general, the blood concentration reaches a stable state after 8 days of administration [21]. We found one meta-analysis [22] that only reported on the effect of aprocitentan on treating hypertension during our search in the abovementioned databases. Although this meta-analysis [22] included eight clinical studies, only the data from two clinical studies were used for the meta-analysis, and no analysis was performed for the other six studies. Therefore, the meta-analysis [22] only incorporated data from two clinical studies. In comparison to the mentioned meta-analysis [22], our study has several advantages: (1) Our study not only analyzed the efficacy of the aprocitentan but also reported on its safety issues; (2) our study divided the aprocitentan dosage into three subgroups (high, medium, low) for a more comprehensive and intuitive demonstration of the impact of dosage on blood pressure reduction. It was previously confirmed that aprocitentan is not an absolute concentration-dependent drug, whereby, in a high-dose subgroup analysis, aprocitentan cannot effectively reduce msSBP; (3) our study not only analyzed the effects of the aprocitentan on msSBP and msDBP but also examined its effects on maSBP and maDBP; (4) our study conducted a meta-analysis of the data from five RCTs, including a larger sample size, which enhances the robustness and persuasiveness of the results.

Treatment-resistant hypertension (TRH) refers to patients with elevated blood pressure, while 24-hour blood pressure is under treatment with three drugs, one being a diuretic [23], and it is currently estimated to be less than 10% in patients undergoing hypertension treatment [24]. However, the risk of cardiovascular events in patients with TRH is 47% [25]. Currently, the clinical application scope and therapeutic effect of traditional renin–angiotensin–aldosterone system (RAAS) antagonists and renal denervation for patients with TRH remain limited [26, 27]. Aprocitentan can significantly reduce blood pressure and has been shown to have additive effects alongside RAAS blockers [21]. Additionally, the mechanism of action of aprocitentan in blocking ET-1 may help lower blood pressure in patients with resistant hypertension and provide more effective cardiovascular protection [20, 28]. However, these findings have not been fully confirmed and require further investigation. Although clinical trials investigating the effect of aprocitentan on blood pressure are not numerous, they have shown encouraging results in decreasing blood pressure [17, 18, 20]. Especially for TRH, aprocitentan shows significant antihypertensive effects and has good safety in patients with chronic kidney disease and moderate liver dysfunction [21, 29]. Therefore, when hypertension cannot be effectively controlled using current treatment methods, aprocitentan represents a novel, effective, and well-tolerated treatment. An additional benefit of aprocitentan is its ability to reduce albuminuria, which may provide renal protection, especially in patients with hypertension and concomitant kidney disease [30].

Although our meta-analysis results indicate that aprocitentan did not cause any SAEs warranting special attention, notable adverse events such as peripheral edema and anemia were observed [17]. Aprocitentan, as a dual endothelin receptor antagonist, is likely to have drug-related adverse events due to its mechanism of action. Studies have demonstrated that ETA-selective receptor antagonists and dual ETA/ETB receptor antagonists can cause fluid retention, which is regarded to be a characteristic side effect of endothelin receptor antagonists (ERAs) [20, 31, 32, 33]. In addition, a mechanistic study in healthy subjects on a high sodium diet showed that aprocitentan induces a moderate (i.e., less than 1 kg) but statistically significant increase in body weight, which could suggest fluid retention [18]. Additionally, Schlaich *et al*. [17] found that aprocitentan may cause anemia. Therefore, when using aprocitentan, doctors should pay attention to whether patients develop fluid retention or anemia and intervene promptly to prevent the occurrence of related AEs such as peripheral edema, pulmonary edema, and heart failure [18, 34].

This study has several limitations. Firstly, although we searched a sufficient number of databases, there may still be studies included in geographically based databases that still need to be included. However, the very small number of studies not included will not affect the results of this study. Secondly, the included studies have brief treatment periods, and there is a lack of sufficient safety evaluation data for the long-term use of aprocitentan in treating hypertension. Thirdly, we performed a subgroup analysis, but several outcomes still had a high heterogeneity. This may be due to the differences in aprocitentan dosages, patient numbers, and treatment durations in the included studies. These factors increase the uncertainty of the clinical outcomes, potentially weakening the robustness of the analysis. Finally, this study strictly adhered to the inclusion and exclusion criteria, and ultimately, only five relatively small studies were included. Nevertheless, there were differences in the baseline levels among the included studies. One study [17] included patients who had received standardized treatment for hypertension, while others [18, 19, 20, 21] included patients who had not received other medication treatments. Further research and larger sample sizes are still needed to confirm the efficacy and safety of aprocitentan, such as longer follow-up periods, larger sample sizes, and multicenter studies.

## 5. Conclusions

The results of this study confirm that aprocitentan can significantly reduce blood pressure, as evidenced by significant effects on msSBP, msDBP, maSBP, and maDBP. Additionally, we did not observe any risks of AEs and SAEs following aprocitentan treatment. However, it is worth noting that these results lack support from large-scale studies, and it is necessary to conduct large RCTs in the future to confirm the efficacy and safety of aprocitentan.

## Availability of Data and Materials

The original data presented in the study are included in the article/supplementary material, further inquiries can be available from the corresponding author upon reasonable request.

## Supplementary Material

Supplementary material associated with this article can be found, in the online version, at https://doi.org/10.31083/RCM25909.
