# Supplementary material for: Efficacy and Safety of Aprocitentan in the Treatment of Hypertension: A Meta-Analysis of Evidence from Randomized Controlled Trials
Source: Rev Cardiovasc Med. 2025 Jan 20;26(1):25909. doi: 10.31083/RCM25909 (PMC11759960; doi:10.31083/RCM25909)
Supplement: Supplementary file 1 [file 2153-8174-26-1-25909-s1.zip › Supplementary Text 1.docx]

**Pubmed**

#1 Hypertension*[Title/Abstract]

#2 " Aprocitentan"[Supplementary Concept]

#3 ACT-132577[Title/Abstract] OR Aprocitentan[Title/Abstract] OR Tryvio[Title/Abstract]

#4 #2 OR #3

#5 "Randomized Controlled Trials as Topic"[Mesh] OR "Randomized Controlled Trial"[Publication Type] OR "Equivalence Trial"[Publication Type] OR "Pragmatic Clinical Trial"[Publication Type] OR "Equivalence Trials as Topic"[Mesh] OR "Intention to Treat Analysis"[Mesh] OR "Pragmatic Clinical Trials as Topic"[Mesh] OR "Single-Blind Method"[Mesh] OR "Random Allocation"[Mesh] OR "Double-Blind Method"[Mesh] OR "Random Allocation"[Mesh] OR "Adaptive Clinical Trial" [Publication Type] OR "Adaptive Clinical Trials as Topic"[Mesh] OR "Clinical Trials, Phase II as Topic"[Mesh] OR "Clinical Trials, Phase III as Topic"[Mesh] OR "Clinical Trials, Phase IV as Topic"[Mesh]

#6 #1 AND #4 AND #5

**Embase**

#1 'randomized controlled trial'/exp OR 'controlled clinical trial'/exp OR 'randomization'/exp OR 'randomized':ab,ti OR 'randomised':ab,ti OR 'randomly':ab,ti OR 'clinical trial (topic)'/exp OR 'placebo'/exp OR 'placebo':ab,ti OR 'trial':ab,ti

#2 'animal'/exp NOT 'human'/exp

#3 #1 NOT #2

#4 'aprocitentan'/exp OR 'ACT-132577':ab,ti OR 'ryvio':ab,ti

#5 #3 AND #4

#6 hypertension:ab,ti

#7 #5 AND #6

**Cochrane Central Register of Controlled Trials**

#1 MeSH descriptor: [hypertension] explode all trees

#2 ("aprocitentan"):ti,ab,kw OR ("ACT-132577"):ti,ab,kw OR ("tryvio"):ti,ab,kw

#3 #1 AND #2

**Clinical Trials.gov**

Condition or disease: hypertension

Intervention/Treatment: aprocitentan or ACT-132577 or tryvio
